# Identification of Novel Hypothalamic MicroRNAs as Promising Therapeutics for SARS-CoV-2 by Regulating ACE2 and TMPRSS2 Expression: An In Silico Analysis

**DOI:** 10.3390/brainsci10100666

**Published:** 2020-09-25

**Authors:** Debasmita Mukhopadhyay, Bashair M. Mussa

**Affiliations:** 1 Biomedical & Chemical Engineering Department, American University of Sharjah, Sharjah 26666, UAE; debasmitag2009@gmail.com; 2 Basic Medical Science Department, College of Medicine, University of Sharjah, Sharjah 27272, UAE

**Keywords:** COVID-19, SARS-CoV-2, hypothalamic circuits, miRNA, ACE2, TMPRSS2

## Abstract

Background: Neuroinvasion of severe acute respiratory syndrome coronavirus (SARS-CoV) is well documented and, given the similarities between this virus and SARS-CoV-2, it seems that the neurological impairment that is associated with coronavirus disease 2019 (COVID-19) is due to SARS-CoV-2 neuroinvasion. Hypothalamic circuits are exposed to the entry of the virus via the olfactory bulb and interact centrally with crucial respiratory nuclei. Hypothalamic microRNAs are considered as potential biomarkers and modulators for various diseases and future therapeutic targets. The present study aims to investigate the microRNAs that regulate the expression of hypothalamic angiotensin-converting enzyme 2 (ACE2) and transmembrane serine protease 2 (TMPRSS2), essential elements for SARS-CoV-2 cell entry. Methods: To determine potential hypothalamic miRNAs that can directly bind to ACE2 and TMPRSS2, multiple target bioinformatics prediction algorithms were used, including miRBase, Target scan, and miRWalk2.029. Results: Our in silico analysis has revealed that, although there are over 5000 hypothalamic miRNAs, around 31 miRNAs and 29 miRNAs have shown binding sites and strong binding capacity against ACE2 and TMPRSS2, respectively. Conclusion: These novel potential hypothalamic miRNAs can be used to identify new therapeutic targets to treat neurological symptoms in COVID-19 patients via regulation of ACE2 and TMPRSS2 expression.

## 1. Introduction

Over the last two decades, multiple waves of coronavirus epidemics have kept the whole world under a stressful situation due to the life-threatening nature of the infection [1]. Previous experiences of severe acute respiratory syndrome coronavirus (SARS-CoV) in 2002, Middle East respiratory syndrome (MERS) in 2012, and the latest novel SARS-CoV-2 in December 2019 have suggested that these infections were transmitted from animals to human [2]. The recent SARS-CoV-2 (coronavirus disease 2019, COVID-19) outbreak devastated the scientific community with unexpected severity and fast spread.

It is noteworthy that SARS-CoV-2 shares almost 80% identical sequences with SARS-CoV [3]. Moreover, SARS-COV-2 also attaches to angiotensin-converting enzyme 2 (ACE2) using the same receptor as SARS-CoV [4]. Additionally, it was observed that ACE2 needs transmembrane serine protease 2 (TMPRSS2) for priming of the viral spike protein. Numerous clinical reports have shown that patients with COVID-19 experienced multiple neurological symptoms such as headaches, vomiting, and nausea, indicating the involvement of the central nervous system (CNS) [5,6,7,8]. Emerging evidence suggests the involvement of brain ACE2 expression in the pathogenesis of COVID-19; therefore, it is of a great interest to explore the neurological effects of SARS-CoV-2 and the associated clinical manifestations in patients with COVID-19 [9]. Hypothalamic circuits are exposed to the entry of the virus via the olfactory blub and interact centrally with the crucial respiratory nuclei, and reach out beyond the CNS to the pituitary and adrenal gland to maintain homeostasis during viral infection [10,11,12]. Therefore, impaired hypothalamic circuits might play a crucial role in the development of neurological manifestations associated with COVID-19 [13,14].

MicroRNAs (miRNAs) are small nucleotides having a length of 22–25 nt and control the regulation of gene expression at the translational level with selectivity for interaction with a single gene or several target genes [15]. In contrast to siRNA or lncRNAs, miRNA-targeted therapy can influence not only a single gene, but entire cellular pathways and processes [16,17,18,19]. It is also possible to supplement down-regulated or non-functional miRNAs by synthetic oligonucleotides, as well as alleviate the effects caused by overexpression of malignant miRNAs through artificial antagonists, oligonucleotides, or small molecules [20]. A current study has exposed that miRNA as an epigenetic regulator can differentiate between SARS-CoV and SARS-CoV-2 and can also reveal the enigmatic science behind the pathogenicity and diverse clinical characteristics of the pandemic of COVID-19 [21]. miRNAs can be an essential antiviral tool not only to fight against the viruses, but also to trigger the innate and adaptive immune system [22]. In addition, miRNAs can indirectly influence the viral propagation and, by having non-antigenic properties, can also modulate cellular pathways without manipulating the host immune response [23,24]. Another recent study has demonstrated that complementary miRNA (cc-miRNA) complexes were predicted to bind and inhibit the translation of corona viral proteins and the replication of SARS-CoV-2, and MERS-CoV genomes [25]. Moreover, miRNA-200c-3p has been found to play a crucial role in acute respiratory distress syndrome and considered as a potential candidate for SARS-CoV-2-related investigations [26].

Most importantly, miRNAs have been considered as potential biomarkers and diagnostic agents for brain tumors including glioblastoma [27]. In addition, the involvement of miRNAs as crucial molecules to investigate the pathogenesis of Alzheimer’s disease (AD) has recently received much attention, and there is developing evidence that miRNAs are promising biomarkers for AD [28]. Furthermore, a strong association has been suggested between miRNAs and the mechanisms of pathogenesis of Parkinson’s disease (PD), which is the second most common neurodegenerative disease. Therefore, microRNAs are considered as a potential alternate therapeutic target to control the progression of PD [29].

The CNS is a prominent site of miRNA expression and several miRNAs are expressed and enriched in different regions within the hypothalamus [30]. miRNAs have recently been shown to be important regulators of various hypothalamic functions [31,32,33,34]. In addition, a recent study has revealed that the hypothalamus is a pivot for understanding the nature of SARS-CoV-2 brain infection [35]. An extensive number of studies revealed multiple roles of different miRNAs directly or indirectly involved in regulating hypothalamic genes. Among them over-expression of miR-200a [36], enriched miR-7a [37], and up-regulation of miR-7b [38], have been extensively studied in order to design nucleic acid-based novel therapeutic strategy against hypothalamus related disorders. Moreover, a high-throughput screening study was able to identify the expression pattern of additional hypothalamic sets of miRNAs, including let-7a, miR-9, miR-30e, miR-132, miR-145, miR-200a, and miR-218, which were altered by caloric restriction and/or a high-fat diet [39,40]. Moreover, association of miR-96/182/183 cluster, miR-141/200c cluster, miR-200a/200b/429 cluster [41], miR-142 and miR-376c [42], microRNA 381/Hes1 [43], miR-361-3p [44], and miR-219 [45] was linked with the development of hypothalamic abnormalities in various studies. Furthermore, recent studies found that hypothalamic miR-141 [46], miR-326 [47], miR-34b, miR-326, miR-432, miR-548c-3p, miR-570, miR-603 [48], miR-488, miR-144, miR-542-5p, miR-421, miR-376b-5p [49], miR-543-5p [50] [51], and miR-363-3p [52] are potential contributors in different neurodegenerative diseases.

The crystal structure of SARS-CoV-2 has a core and receptor-binding domain (RBD), which can recognize hACE2 for its initial entry inside the body [4]. The ubiquitous nature and higher expression of hACE2 throughout the body was the main reason for the fast spread of the virus [53]. However, some recent data have revealed the neurological manifestations in some COVID-19 patients with symptoms such as anosmia, ageusia, and encephalopathy, which further confirmed the neuro-invasion of the virus [3]. The human brain is found to express a large amount of hACE2, thus it is considered as one of the major entry points for SARS-CoV-2. Earlier reports involving SARS-CoV and MERS-CoV elucidated the infiltrating ability against the brain of transgenic mice upon intranasal administration, which was further reported to affect the brain stem and thalamus [53].

ACE2 serves as a negative regulator of renin-angiotensin aldosterone system (RAAS) and cleaves angiotensin (Ang) I to generate the inactive Ang-(1-9) peptide, which can then be converted to Ang-(1-7) by ACE [54]. ACE2 can directly metabolize Ang II to generate the beneficial heptapeptide Ang-(1-7), whose actions are often opposite to those attributed to the Ang II and its type 1 receptor (AT1R) signaling [55]. Apelin, a second catalytic substrate for ACE2, has powerful positive inotropic actions and vasodilatation in an endothelium- and nitric oxide- (NO-) dependent way [56]. Intriguingly, recent studies have demonstrated that there is a link between the ACE2/Apelin signaling and miRNAs in the pathogenesis of hypertension [57]. In 2013, Kohlstedt K et al. demonstrated that AMP-activated protein kinase regulates endothelial cell angiotensin-converting enzyme (ACE) expression via p53 and the post-transcriptional regulation of microRNA-143/145 [58]. In 2014, another study revealed that mechanical stretch suppresses the expression of miR-145 by up-regulating ACE in vascular smooth muscle [59]. Further, it was been demonstrated that Angiotensin II-regulated microRNA 483-3p directly targets multiple components of the rennin-angiotensin system [60]. In the same year, Lambert et al. established that miR-421, responsible for the development of thrombosis, was able to down-regulate ACE2 expression [61]. Furthermore, elevated level of miR-27a coupled with reduced miR-143 expression was found to be associated with exercise-induced hypertensive rats (SHR) by directly targeting ACE2 [62]. A very recent study demonstrated that miRNA let-7b promotes the development of hypoxic pulmonary hypertension by targeting ACE2 directly [59]. However, detailed studies related to involvement of miRNA with ACE2 expression associated with hypothalamus are yet to be conducted.

Another important aspect to comprehend the mechanistic detail of SARS-CoV-2 infection is the involvement of TMPRSS2, a cell-surface protein that is expressed by epithelial cells of several tissues [63]. As an initial step to enable the host cell entry, the viral hemagglutinin protein attaches to human angiotensin-converting enzyme 2 (hACE2), followed by cleavage of hemagglutinin to activate internalization of the virus, which is dependent on proteases on the host cell, particularly TMPRSS2 [63]. Moreover, not only SARS-CoV-2, but also other types of coronaviruses and influenza viruses depend on TMPRSS2 for viral activation and cell entry, including SARS-CoV, the agent responsible for the 2003 SARS outbreak, as well as influenza H1N1, the agent responsible for the 1918 and 2009 influenza pandemics [64,65]. These examples highlight the central role of TMPRSS2 in the pathogenesis processes of coronaviruses and influenza viruses. TMPRSS2 was first identified to be highly expressed in prostate cancer [66]. Notably, the TMPRSS2 gene is a partner in one of the most common gene fusion events in solid tumors, namely the ETS (E26 transformation-specific or Erythroblast Transformation Specific genes) family of oncogenic transcription factors, most commonly ERG (ETS-related gene). However, the ERG gene is consequently controlled by androgen receptor signaling and expressed highly in prostate cancers harboring the TMPRSS2–ERG fusion. TMPRSS2–ERG fusions are being pursued as genomic biomarkers to predict treatment responses in prostate cancer (PCa). Taking into account these findings along with the potential novel treatments for COVID-19, such as manipulation of ACE2 receptor, the existing data on TMPRSS2-targeted treatments can be considered as a promising route of therapeutics that can be further investigated. Successful biomarker development depends on the frequency of TMPRSS2 fusions and miR-25-miR-93-miR-106b cluster, and miR-152 and miR-423-5p were expressed differentially in TMPRSS2 fusion-negative disease in African American men cohort [67].

In our present study, we have shortlisted the potential hypothalamic miRNAs based on in silico analysis using different microRNA target prediction software with a focus on expression of ACE2 and TMPRSS2.

## 2. Materials and Methods

To determine potential miRNAs that can directly bind to ACE2 and TMPRSS2, multiple target bioinformatics prediction algorithms were used including miRbase (http://www.mirbase.org), miRAnda (www.http://microRNA.org), Target scan (http://www.targetscan.org/vert_72), and miRWalk2.029 (http://zmf.umm.uni-heidelberg.de/apps/zmf/mirwalk2/cellpub.html). These computational approaches work in association with miRNA and mRNA nucleotide sequences in a special format that governs the binding characteristics between the exact beginning sequence followed by 7 to 11 nucleotides miRNA binding site on the mRNA, which is also known as ‘seed match’. The location of the miRNA binding site within the specified ACE2 and TMPRSS2 gene can be either untranslated region (3′UTR, 5′UTR) or coding sequences (CDS). Furthermore, it is a Gibb’s free energy that is used as a measure of stability of miRNA structure by many tools. When a miRNA binds to the target mRNA, resulting in a stable structure, it is considered as the most likely target of that miRNA. The reactions with more negative delta-G are less reactive, and thus have more stability. The hybridization of miRNA with its target mRNA provides information about the high and low free energy regions and delta-G predicts the strength of bonding between the miRNA and its target mRNA. The interaction free energy (∆G, kJ/mole), which further determines the collaboration pattern among nucleotides of miRNAs and mRNAs, plays a crucial role during the intercellular gene silencing phenomenon, which needs to be critically selected. Furthermore, the ratio of ΔG/ΔGm (%) was determined for each binding site, where ΔGm is equal to the free energy binding of miRNA with its full complementary nucleotide sequence. Both the miRnada and MirTarget program found hydrogen bonds between adenine (A) and uracil (U), guanine (G) and cytosine (C), G and U, and A and C. It has been observed that each prediction analysis tool is equipped with different algorithms, and it is difficult to know which tool is the best choice for their study. In this study, we shortlisted only those miRNAs that were common in the three softwares based on the following characteristics: having 7–8 seed match, GU Wobble seed match, which calculates the chances of a G pairing with a U instead of C; position contribution; seed pairing stability; target-site abundance, which determines the number of sites occurring in the 3′-UTR; local AU, which flanks in the corresponding seed region; and 3′-compensatory pairing, which is the pairing region (12–17 nts) in which the base pairs match with miRNA nucleotides.

## 3. Results

### 3.1. Identification of Potential Hypothalamic miRNAs Involved in Regulation of ACE2

Initial analysis has revealed more than 5000 hypothalamic miRNAs associated with these two genes, ACE2 and TMPRSS2, directly or through different pathways.

In our present study, we have taken the approach of altering the expression of ACE2 by miRNAs. Our preliminary in silico miRNA prediction analysis revealed almost 31 miRNAs having potential binding efficiency against ACE2 genes that can be linked with the hypothalamus. These miRNAs might enhance or suppress the expression of ACE2 by direct binding or through some other pathways, revealing a potential target for therapeutic strategy for SARS-CoV-2-2 infection. Table 1 shows a list of novel potential candidate miRNAs against ACE2 gene that were not studied previously.

### 3.2. Identification of Potential Hypothalamic miRNAs Involved in Regulation of TMPSS2

Our preliminary in silico miRNA prediction analysis revealed almost 29 miRNAs having potential binding efficiency against TMPRSS2 gene and they can be linked with hypothalamus. Table 2 shows a list of novel potential candidate miRNAs against TMPRSS2 gene which were not studied previously.

## 4. Discussion

SARS-CoV-2 is not only a new version of the SARS-CoV virus that causes the common cold; it has a number of unusual and sometimes alarming features including severe neurological complications [68]. As of end of August 2020, more than 14 million confirmed cases of SARS-CoV-2 infection have been reported globally, with a mortality rate of ~3%. In addition, several studies have highlighted the neurological manifestations associated with SARS-CoV-2 including hyposmia, headache, and consciousness [8]. Unfortunately, there are no effective antiviral drugs existing against SARS-CoV-2. Therefore, there is an extreme urgency to develop novel therapies for COVID-19.

Recent studies involving human brain gene-expression analyses revealed that the hypothalamus and associated regions express hACE2 and hTMPRSS2, which mediate SARS-CoV-2 cellular entry, in correlation with several genes and pathways that may be involved in the disturbance of physiological functions and viral pathogenesis [35]. These findings suggest that the hypothalamus could be a central region for SARS-CoV-2 brain invasion through multiple routes. While in humans, more than 70% of the genome is transcribed to RNA, only ~2% of these transcripts are translated into proteins coding genes and finally proteins. The rest of the transcripts are demarcated as noncoding RNAs, including microRNAs, which typically function post-transcriptionally to control the gene expression [69]. Various RNA viruses mimic or block the binding between a host miRNA and its target transcript, a phenomenon mediated by the miRNA seed site at the 5′ end of miRNA [70]. In past days, respiratory viral infections caused by influenza, rhinovirus, adenovirus, RSV, and coronaviruses can be related to aberrant host miRNA expression, and their effect on the host can results in cell apoptosis, inhibition of immunologic pathways, and downregulation of host antiviral responses [21]. To date, many studies have tried to investigate different signaling pathways of SARS-CoV-2 with concomitant association of miRNA. However, it is not fully understood with respect to controlling the expressions of genes that are associated with neurological impairment.

In our present study, we utilized bioinformatic approaches to investigate the interaction network of SARS-CoV-2 and the human hypothalamus, which are linked with miRNAs. It is crucial to provide a structured prediction of the potential hypothalamic miRNAs that are involved in the pathogenesis of neurological impairment associated with SARS-CoV-2. We conduct a comprehensive analysis of the most significant gene sets (hACE2 and hTMPRSS2) using miRBase, Target scan, and miRWalk2.029 algorithm. This analysis resulted in a network of miRNA–mRNA interactions illustrated in Table 1; Table 2. Various mechanisms based on miRNA-mediated regulation of viruses have been reported. We searched for miRNAs that shared 100% identity of the 8 mer seed region against respective genes. Our present in silico analysis is able to provide us the possibility to deliver novel miRNA networks targeting the two major hypothalamic genes (hACE2 and hTMPRSS2) and their signaling pathways in response to SARS-CoV-2 infection. Finally, we believe that the outcomes of the present study represent an initial step to redirect the attention toward miRNAs as vital contributors to development of diagnostics, biomarkers, and therapeutic targets for COVID-19.

## 5. Conclusions

Diagnostic and interventional medicine has evolved to include miRNAs, which can be used to identify regulatory networks of new and unconventional therapeutic targets against SARS-CoV-2 infection. Moreover, the use of antisense oligonucleotides to block viral genome is considered as the most important way toward new therapeutic strategies. In our present study, we have summarized the potential miRNAs that may be involved in the control of SARS-CoV-2 neuroinvasion via regulating the expression of hypothalamic hACE2 and hTMPRSS2 genes.

## Figures and Tables

**Table 1:** List of miRNAs having strong binding potential against hACE2. ACE2, angiotensin-converting enzyme 2.

	miRNA	Sequence (5′ to 3′)
1	hsa-miR-106b-5p	TAAAGTGCTGACAGTGCAGAT
2	hsa-miR-130a-3p	CAGTGCAATGTTAAAAGGGCAT
3	hsa-miR-141-3p	TAACACTGTCTGGTAAAGATGG
4	hsa-miR-148a-3p	TCAGTGCACTACAGAACTTTGT
5	hsa-miR-149-5p	TCTGGCTCCGTGTCTTCACTCCC
6	hsa-miR-200a-3p	TAACACTGTCTGGTAACGATGT
7	hsa-miR-200b-3p	TAATACTGCCTGGTAATGATGA
8	hsa-miR-200c-3p	TAATACTGCCGGGTAATGATGGA
9	hsa-miR-203a-3p	GTGAAATGTTTAGGACCACTAG
10	hsa-miR-300	TATACAAGGGCAGACTCTCTCT
11	hsa-miR-326	CCTCTGGGCCCTTCCTCCAG
12	hsa-miR-329-3p	AACACACCTGGTTAACCTCTTT
13	hsa-miR-330-3p	GCAAAGCACACGGCCTGCAGAGA
14	hsa-miR-362-3p	AACACACCTATTCAAGGATTCA
15	hsa-miR-371a-5p	AAGTGCCGCCATCTTTTGAGTGT
16	hsa-miR-376a-3p	AAGTGCCGCCATCTTTTGAGTGT
17	hsa-miR-376b-3p	ATCATAGAGGAAAATCCACGT
18	hsa-miR-376c-3p	AACATAGAGGAAATTCCACGT
19	hsa-miR-381-3p	TATACAAGGGCAAGCTCTCTGT
20	hsa-miR-421	ATCAACAGACATTAATTGGGCGC
21	hsa-miR-429	TAATACTGTCTGGTAAAACCGT
22	hsa-miR-494-3p	TGAAACATACACGGGAAACCTC
23	hsa-miR-495-3p	AAACAAACATGGTGCACTTCTT
24	hsa-miR-511-3p	AATGTGTAGCAAAAGACAGA
25	hsa-miR-543	AAACATTCGCGGTGCACTTCTT
26	hsa-miR-548m	CAAAGGTATTTGTGGTTTTTG
27	hsa-miR-2113	ATTTGTGCTTGGCTCTGTCAC
28	hsa-miR-3611	TTGTGAAGAAAGAAATTCTTA
29	hsa-miR-3976	TATAGAGAGCAGGAAGATTAATGT
30	hsa-miR-4778-3p	TCTTCTTCCTTTGCAGAGTTGA
31	hsa-miR-5197-3p	AAGAAGAGACTGAGTCATCGAAT

**Table 2:** List of miRNAs having strong binding potential against hTMPRSS2. TMPRSS2, transmembrane serine protease 2.

	miRNA	Sequence
1	hsa-let7a-5p	TGAGGTAGTAGGTTGTATAGTT
2	hsa-let7b-5p	TGAGGTAGTAGGTTGTGTGGTT
3	hsa-let7c-5p	TGAGGTAGTAGGTTGTATGGTT
4	hsa-let7d-5p	AGAGGTAGTAGGTTGCATAGTT
5	hsa-let7e-5p	TGAGGTAGGAGGTTGTATAGTT
6	hsa-let7f-5p	TGAGGTAGTAGATTGTATAGTT
7	hsa-let7g-5p	TGAGGTAGTAGTTTGTACAGTT
8	hsa-let7i-5p	TGAGGTAGTAGTTTGTGCTGTT
9	hsa-miR-7-5p	TGGAAGACTAGTGATTTTGTTGTT
10	hsa-miR-25-3p	CATTGCACTTGTCTCGGTCTGA
11	hsa-miR-32-5p	TATTGCACATTACTAAGTTGCA
12	hsa-miR-92a-3p	TATTGCACTTGTCCCGGCCTGT
13	hsa-miR-92b-3p	TATTGCACTCGTCCCGGCCTCC
14	hsa-miR-98-3p	CTATACAACTTACTACTTTCCC
15	hsa-miR-153-5p	TTGCATAGTCACAAAAGTGATC
16	hsa-miR-182-5p	TTTGGCAATGGTAGAACTCACACT
17	hsa-miR-183-5p	TATGGCACTGGTAGAATTCACT
18	hsa-miR-214-3p	ACAGCAGGCACAGACAGGCAGT
19	hsa-miR-363-3p	AATTGCACGGTATCCATCTGTA
20	hsa-miR-367-3p	AATTGCACTTTAGCAATGGTGA
21	hsa-miR-448	TTGCATATGTAGGATGTCCCAT
22	hsa-miR-494-3p	TGAAACATACACGGGAAACCTC
23	hsa-miR-511-3p	AATGTGTAGCAAAAGACAGA
24	hsa-miR-4458	AGAGGTAGGTGTGGAAGAA
25	hsa-miR-4500	TGAGGTAGTAGTTTCTT
26	hsa-miR-4778-3p	TCTTCTTCCTTTGCAGAGTTGA
27	hsa-miR-4796-5p	TGTCTATACTCTGTCACTTTAC
28	hsa-miR-5197-5p	CAATGGCACAAACTCATTCTTGA
29	hsa-miR-6864-3p	GTGAGACTTCTCTCCCTTCAG
